# Clinico-pathological characteristics and treatment outcome in non-small cell lung cancer in Greenland 2015–2020 – a comparison with the cohort from 2004 to 2010

**DOI:** 10.2340/1651-226X.2024.41078

**Published:** 2024-12-17

**Authors:** Dorte S. Nørøxe, Simone Frandsen, Malene M. Clausen, Thomas Irgens, Uka W Geisler, Alice J. Petersen, Seppo W. Langer

**Affiliations:** a Department of Oncology, Copenhagen University Hospital, Rigshospitalet, Denmark; b Department of Medicine, Queen Ingrids Hospital, Nuuk, Greenland; c Department of Clinical Physiology and Nuclear Medicine & Cluster for Molecular Imaging, Copenhagen University Hospital, Denmark; d Department of Clinical Medicine, University of Copenhagen, Denmark

**Keywords:** Non-small-cell lung cancer, lung cancer, Greenland, gene mutation, clinical and pathological

## Abstract

**Background and purpose:**

Lung cancer is the leading cause of cancer-related mortality in Greenland. Since 2004, medical treatment of lung cancer has been available in Greenland. However, both diagnostic work-up and treatment logistics are hampered by the lack of medical services in smaller settlements, the infrastructure, and extreme arctic weather conditions. Clinico-pathological data and assessment of treatment outcome in lung cancer in Greenland have not been carried out since 2015. This study aims to provide an analysis of Greenlandic patients with non-small cell lung cancer (NSCLC) from 2015 to 2020, compared to the cohort from the 2015 study. We focus on diagnostics, patient and treatment characteristics, and survival rates. Additionally, we include new data on treatment-related factors and diagnostic delays.

**Patients/material:**

Clinical-, pathological-, genomic data, tuberculosis status and survival were retrieved from the medical journal.

**Results:**

A total of 163 patients were identified. Survival had improved in stage I, III, and IV, and early-stage disease was more often diagnosed as compared to the 2015 cohort. Molecular alterations and PD-L1 expressing tumors were comparable between Greenlandic and Danish patients. Diagnostic delay was a major concern.

**Interpretation:**

While NSCLC survival in Greenland has improved over the past decade, significant challenges remain. The trend towards diagnosing more stage IA–IIIA patients and the recent improvements in diagnostic and therapeutic options in Greenland are expected to translate into a better prognosis in the coming years. Addressing diagnostic delays and enhancing treatment options are crucial steps toward improving outcomes for NSCLC patients in Greenland.

## Introduction

Greenland has a population of 56,000, with 19,000 residing in the capital, Nuuk, where the national hospital, Queen Ingrid’s Hospital (QIH) is located. Health care services in the towns and settlements along the coast are provided by traveling medical consultants [1, 2]. As part of the Kingdom of Denmark, Greenland’s healthcare system is closely integrated with the Danish system.

Lung cancer is the leading cause of cancer-related deaths in Greenland, with 51 new cases annually, equating to 91 per 100,000 people per year [3, 4]. While the incidence has decreased among men since 2010, it is rising rapidly among women. Since 2004, medical treatment of lung cancer has been available at QIH and approximately 30 patients are referred yearly to QIH. However, both diagnostic work-up and treatment logistics are hampered by the lack of medical services in smaller settlements, a challenging infrastructure, and often extreme arctic weather conditions. Tuberculosis (TB) is endemic in Greenland, and routine TB testing is performed in patients with, for example, abnormal chest X-rays. This testing can potentially contribute to delays in lung cancer diagnosis. Diagnostic procedures and treatments are conducted in accordance with the guidelines established by the Danish Lung Cancer Group, in close collaboration with the departments of oncology, pulmonary medicine, thoracic surgery, nuclear medicine, and pathology at Copenhagen University Hospital, Rigshospitalet in Copenhagen, Denmark [5]. Consequently, the initial pathological work-up, parts of the staging process, as well as all surgical and radiation therapies, are performed at Rigshospitalet.

In recent years, the treatment of non-small cell lung cancer (NSCLC) has improved significantly due to novel targeted treatments and immune checkpoint inhibitors (ICI) [6–11]. Real-life data from a Danish nationwide study revealed that the implementation of ICI has tripled the 3-year survival in metastatic NSCLC from 6 to 18% [12, 13]. Treatment with ICI was approved in Denmark in 2017. Until recently, Greenlandic patients were referred to Rigshospitalet for treatment with ICI, but in 2023, ICI treatment became available at QIH and is now offered to patients on the same indications as in Denmark.

To our knowledge, clinicopathological data and assessment of treatment outcome in lung cancer in Greenland have not been carried out since the description of the 2004–2010 cohort in 2015 [14]. The aim of this study was to perform an updated analysis on the Greenlandic population of NSCLC, focusing on diagnostics, patient and treatment characteristics, and current survival rates. Additionally, this study includes new data on treatment-related factors and diagnostic delays.

## Methods and materials

When a Greenlandic patient is diagnosed with lung cancer, it is registered in the Greenlandic Oncology Registry at QIH. The diagnosis is ideally confirmed through pathology, but in some cases, it may rely solely on radiological findings. We searched the Greenlandic Oncology Registry for lung cancer from 2015 to 2020 and extracted cases with a pathological confirmed diagnosis of NSCLC. Clinical, pathological, and genomic data (including mutational status in *EGFR*, *ROS1*, *BRAF*, and *KRAS*, *ALK* fusions, and PD-L1 status) were extracted from the QIH medical records and the Danish pathology database. The presence of mycobacterium TB was detected by QuantiFERON^®^ TB Gold (QFT) or by sputum analysis. The time from symptom onset to a pathologically confirmed diagnosis, as well as the time from diagnosis to the start of treatment, was registered. The date of symptom onset was patient-reported, for example, the first day of symptoms of NSCLC. In patients with suspicious findings on radiological examination carried out for another indication, the date of radiological examination was used. In cases with unclear onset of symptoms, the date of first contact with the health care system was used. We defined a delayed diagnostic group versus a non-delayed diagnostic group by the median time in days from symptoms to diagnosis.

Overall survival (OS) was calculated from the date of diagnosis to death. The Tumor, Node, Metastasis (TNM) staging system v8 was used. Statistical analyses including two-sided Fishers exact test for TB analyses and diagnostic delay, and Kaplan-Meier and Cox Proportional Hazard for survival analyses using SPSS v.29.0.1.0. Time of data cut-off was 21.02.2024. The study was approved by the Greenlandic Ethical Committee (KVUG 2021-27), the Head of Medical Department at QIH, and the hospital management of QIH.

## Results

### Patient characteristics

**Figure 1:**
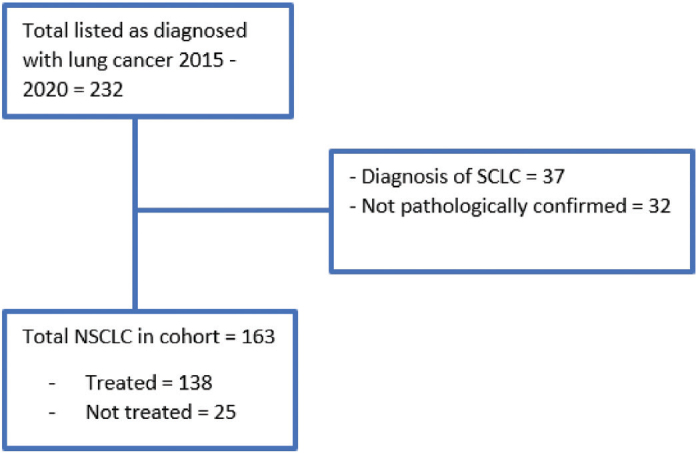
Consort diagram. The Greenlandic Oncology Registry had 232 patients listed with the diagnosis of lung cancer in the period of 2015–2020. The diagnosis of lung cancer included non-small-cell lung cancer (NSCLC), small-cell lung cancer (SCLC), mesothelioma, and more. After reviewing the medical records for patients diagnosed with NSCLC only, the cohort was 163 patients.

Patient characteristics are listed in Table 1. In the period of 2015–2020, and according to the Greenlandic Oncology Registry, 232 patients were categorized as having lung cancer. Small-cell lung cancer (SCLC) was diagnosed in 37 of the patients, but in 32/232 cases (14%), the diagnosis was not pathologically confirmed. Reasons for lack of histological confirmation were usually due to a general poor condition of the patient, making further examinations and treatment impossible. Hence, we identified 163 patients with pathologically confirmed NSCLC (Figure 1). The median age at diagnosis was 65 years with a male: female ratio of 3:2. Most patients were in performance status (PS) 0–1 (72%), and information was missing for eight patients (5%). The median time from symptoms to diagnosis was 85 days (range 13–1,249). The median time from diagnosis to initiation of treatment was 18 days (range 0–117). Current smokers were identified in 123 patients (76%), and three patients were never smokers. The median smoking pack year was 25.

**Table 1:** Patient characteristics, *N* = 163.

Variables	All, *N* = 163	Treated, *N* =138	Not treated, *N* = 25
**Age (median)**	65 (40–83)	64 (40–79)	69 (52–83)
**Female sex, *N* = 163, (%)**	62 (38.0)	55 (39.9)	7 (28.0)
**Performance status at initiation of oncological treatment, (%)**			
**- 0–1**	117 (71.8)	115 (83.3)	2 (8.0)
**- 2**	14 (8.6)	14 (10.1)	0 (0)
**- 3–4**	24 (14.7)	1 (0.7)	23 (92.0)
**- Missing**	8 (4.9)	8 (5.8)	0 (0.0)
**Tuberculosis sputum, tested, (%)**	142 (87.1)	122 (88.4)	20 (80.0)
**- Positive**	3 (2.1)	3 (2.5)	0 (0)
**- Negative**	139 (97.9)	119 (97.5)	20 (100)
**Tuberculosis QuantiFERON^®^ TB Gold (QFT), tested, (%)**	120 (73.6)	104 (75.4)	16 (64.0)
**- Positive**	32 (26.7)	28 (26.9)	4 (25.0)
**- Negative**	88 (73.3)	76 (73.1)	12 (75.0)
**Smoking status, (%)**			
**- Median smoking pack years (range)**	25 (5–140)	30 (5–140)	25 (10–40)
**- Current**	123 (75.5)	109 (79.0)	14 (56.0)
**- Former**	32 (23.2)	26 (18.8)	6 (24.0)
**- Never**	3 (3.7)	2 (1.5)	1 (4.0)
**- Missing**	5 (3.1)	1 (0.7)	4 (16.0)
**Histology, (%)**			
**- Adenocarcinoma**	58 (35.6)	46 (33.3)	12 (48.0)
**- Squamous cell carcinoma**	100 (61.3)	88 (63.8)	12 (48.0)
**- NSCLC, NOS**	5 (3.1)	4 (2.9)	1 (4.0)
**Stage I–IV, all, (%)**			
**- I**	21/163 (12.9)	20 (14.5)	1 (4.0)
**- II**	11/163 (6.7)	11 (8.0)	0 (0)
**- III**	40/163 (24.5)	39 (28.3)	1 (4.0)
**- IV**	83/163 (50.9)	60 (43.5)	23 (92.0)
**- Missing**	8/163 (4.9)	8 (5.8)	0 (0)
**Stage I–IV, ADC, (%)**	*N* = 58	*N* = 46	*N* = 12
**- I**	11/58 (19.0)	10 (21.7)	1 (8.3)
**- II**	5/58 (8.6)	5 (10.9)	0 (0)
**- III**	11/58 (19.0)	11 (23.9)	0 (0)
**- IV**	31/58 (53.4)	20 (43.5)	11 (91.7)
**- Missing**	0/58 (0)	0 (0.0)	0 (0)
**Stage I–IV, SCC, (%)**	*N* = 100	*N* = 88	*N* = 12
**- I**	10/100 (10.0)	10 (11.4)	1 (8.3)
**- II**	5/100 (5.0)	5 (5.7)	0 (0)
**- III**	29/100 (29.0)	28 (31.8)	0 (0)
**- IV**	48/102 (48.0)	37 (42.0)	11 (91.7)
**- Missing**	8/100 (8.0)	8 (9.1)	0 (0)
**Stage IA–IV, all, (%)**	*N* = 163	*N* = 138	*N* = 25
**- IA-B**	21/163 (12.9)	20 (14.5)	1 (4.0)
**- IIA-B**	11/163 (6.7)	11 (8.0)	0 (0)
**- IIIA**	23/163 (14.1)	23 (16.7)	0 (0)
**- IIIB-C+IV**	100/163 (61.3)	76 (55.1)	24 (96.0)
**- Missing**	8/163 (4.9)	8 (5.8)	0 (0)
**Stage IA–IV, ADC, *N* = 58, (%)**		*N* = 46	*N* = 12
**- IA-B**	11 (19.0)	10 (21.7)	1 (8.3)
**- IIA-B**	5 (8.6)	5 (10.9)	0 (0)
**- IIIA**	7 (12.1)	7 (15.2)	0 (0)
**- IIIB-C+IV**	35 (60.3)	24 (52.2)	11 (91.7)
**- Missing**	0 (0)	0 (0.0)	0 (0)
**Stage IA–IV, SCC, (%)**	*N* = 100	*N* = 88	*N* = 12
**- IA-B**	10 (10.0)	10 (11.4)	0 (0)
**- IIA-B**	5 (5.0)	5 (5.7)	0 (0)
**- IIIA**	16 (16.0)	16 (18.2)	0 (0)
**- IIIB-C+IV**	61 (61.0)	49 (55.7)	12 (100.0)
**- Missing**	8 (8.0)	8 (9.1)	0 (0)
**Mutations, *N* (%)**	At least one analysis was performed in 55 patients	At least one analysis was performed in 45 patients	At least one analysis was performed in 10 patients
**- *EGFR* mutation (all in exon 21, L858R)**	4/51 (7.8)	4/41 (9.8)	0/10 (0.0)
**- *ALK* fusion**	1/55 (1.8)	1/45 (2.3)	0/10 (0.0)
**- *ROS1* mutation**	0/17 (0)	0/14 (0.0)	0/3 (0.0)
**- *BRAF* mutation (V600E)**	1/38 (2.6)	0/31 (0.0)	1/7 (14.3)
**- *KRAS* mutation (G12C)**	8/39 (20.5)	8/32 (25.0)	0/7 (0.0)
**PDL1 status, *N* = 105, (%)**	*N* = 105	*N* = 90	*N* = 15
**- <1%**	45 (42.9)	37 (41.1)	8 (53.3)
**- 1–50%**	36 (34.3)	32 (35.6)	4 (26.7)
**- >50%**	24 (22.9)	21 (23.3)	3 (20.0)
**First treatment given, *N* = 163, (%)**	*N* = 163		
**- Surgery (± chemotherapy)**	29 (17.8)	NA	NA
**- Curative radio therapy (+/- chemotherapy)**	17 (10.4)	NA	NA
**- Systemic treatment (incl chemotherapy, IT, targ tx)**	84 (51.5)	NA	NA
**- Palliative radiotherapy (incl lung, brain, MTS)**	8 (4.9)	NA	NA
**- None**	25 (15.3)	NA	NA
**Radiation therapy anytime in disease course, (%)**	*N* = 138	*N* = 138	
	60 (43.5)	60 (43.5)	NA
**Time from symptoms to diagnosis, days (median [range]),**	*N* = 162	*N* = 137	*N* = 25
	85 (13–1249)	85 (13–1249)	68 (21–417)
**Time from diagnosis to start treatment, days (median [range])**	*N* = 137	*N* = 137	
	18 (0–117)	18 (0–117)	NA

ADC, adenocarcinoma; SCC, squamous cell carcinoma; NSCLC, non-small cell lung cancer; TB: Tuberculosis; NOS: not otherwise specified.

### Stage and pathology

Fifty-eight patients (36%) had adenocarcinoma, 100 (61%) squamous cell carcinoma, and NSCLC-not otherwise specified (NOS) was present in five patients (3%). Early-stage (IA–IIIA) was diagnosed in 34% and late stage (IIIB–IV) in 61%. Disease stages are presented in Table 1. Genomic analyses for one or more alterations were performed in 55 patients (34%). Not all genomic analyses were performed in all patients. Different sequencing panels were used over the years, for example, COBAS4800 or Illumina TruSight panels. Genomic analyses were performed more frequently in patients with adenocarcinoma (50/55 cases) than in squamous cell carcinoma (4/100) and NSCLC-NOS (1/5). The most common genomic alterations detected were *KRAS* G12C mutation in 8/39 (21%), *EGFR* mutation (exon 21, L858R) in 4/51 (8%), and *ALK* fusion and *BRAF* V600E in 1 patient each, respectively. *ROS1* mutation was not detected. PD-L1 expression analysis was performed in 105 cases and was equally distributed in adenocarcinoma and squamous cell carcinoma. PD-L1 <1%, 1–50% and >50% were present in 45 (43%), 36 (34%), and 24 (23%), respectively.

### Treatment

A total of 138 patients (85%) underwent treatment, while 25 patients (15%) did not receive any anticancer treatment. Among the treated patients, 29 (18%) underwent primary surgery, including 9 who had open surgery and 20 who underwent endoscopic (video assisted thoracic surgery) lobectomy or segmentectomy. Adjuvant platin-based chemotherapy was administered when indicated (*n* = 18). Seventeen patients (11%) received curatively intended radiotherapy (RT) at 66 Gy/33 fractions, with or without three cycles of concomitant platin-doublet. Primary palliative medical treatment was administered to 84 patients (52%), with 81 patients receiving carboplatin and vinorelbine, one receiving *EGFR*-targeted therapy with a tyrosine kinase inhibitor (TKI), and two receiving ICI in Denmark. Eight patients (5%) received primary palliative RT alone, including three patients in stages IA, IIIA, and IIIB, respectively. The remaining five patients had stage IV disease.

### Survival

**Figure 2:**
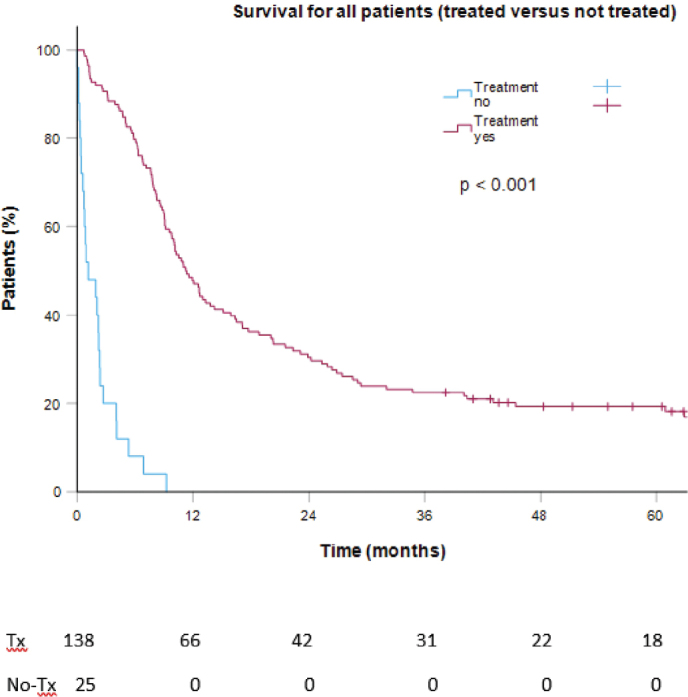
Survival for the full cohort, stratified for patients receiving treatment (tx) versus not receiving treatment (no-tx). *N* = 163.

**Figure 3:**
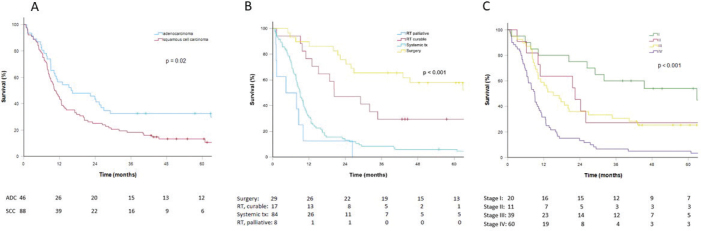
(A) Survival in the treated group, stratified for adenocarcinoma versus squamous cell carcinoma, excluding NSCLC not otherwise specified. *N* = 134. Patients with adenocarcinoma had a better median overall survival (OS) of 15.9 months (95% CI: 2.0–29.8) versus 10.3 months (95% CI: 8.0–12.5) in squamous cell carcinoma. The difference was statistically significant (*p* = 0.02). (B) Survival in the treated group, stratified for treatment. *N* = 138. RT: radiotherapy. RT, curable includes normo-fractionated RT and stereotactic RT ± concurrent chemotherapy. Systemic treatment includes chemotherapy, targeted treatment, and immunotherapy. Surgery includes ± adjuvant chemotherapy. There was a statistically significant difference in median OS between treatments with surgery versus RT curable versus systemic treatment versus RT palliative of 71.7 months (95% CI: 30.7–112.8) versus 20.3 months (95% CI: 1.6–39.1) versus 9.1 months (95% CI: 7.5–10.7) versus 7.9 months (95% CI: 5.1–10.7), respectively (*p* < 0.001). (C) Survival in the treated group, stratified for stage. Patients with stage missing are excluded. *N* = 130. There was a statistically significant difference between stage I versus II, versus III, versus IV, respectively, with a median OS of 62.9 months (95% CI: 12.3–113.5) versus 22.4 months (95% CI: 7.9–36.9) versus 15.1 months (95% CI: 8.2–22.1) versus 9.0 months (95% CI: 7.6–10.4), respectively (*p* < 0.001).

The overall 5-year survival rate was 17%, with a median overall survival (mOS) of 9.6 months. Survival data comparing treated versus untreated patients are shown in Figure 2 and Table 2. Among treated patients with adenocarcinoma versus squamous cell carcinoma, the 5-year survival rates were 33 and 13%, respectively, and with mOS of 15.9 months versus 10.3 months (*p* = 0.02, Figure 3 and Table 2). Patients who underwent intended curative surgery had a 5-year survival rate of 58% and a mOS of 71.7 months, while those receiving curatively intended RT, with or without concomitant chemotherapy, had a 5-year survival rate of 30% and a mOS of 20.3 months. For patients receiving palliative medical treatment, the 5-year survival rate was 6%, with a mOS of 9.0 months. Among those receiving palliative RT, only one patient survived beyond 1 year, with a mOS of 4.4 months. For patients treated at stages I, II, III, and IV (*n* = 130), the 5-year survival rates were 54, 27, 26, and 5%, respectively, with mOS of 62.9, 22.4, 15.1, and 9.0 months, respectively (Figure 3 and Table 2). The 1-, 2- and 5 years survival rates for all treatment groups are displayed in Table 2.

**Table 2:** Median survival, and 1-, 2-, and 5-year survival rates for all patients, and for treated patients divided by histology, treatment given and stage.

Variables	Median, months (95% CI)	1 year survival rate (%)	2 year survival rate (%)	5 year survival rate (%)
**All patients**	9.6	40	26	17
Treated	11.2 (9.3–13.2)	48	30	19
Not treated	1.2 (0.0–3.0)	0	0	0
**Histology – treated**				
Adenocarcinoma	15.9 (2.0–29.8)	56	44	33
Squamous cell carcinoma	10.3 (8.0–12.5)	44	25	13
**Treatment given**				
Surgery	71.7 (30.7–112.8)	86	72.5	58
RT curative	20.3 (6.6–34.1)	76.5	47	29.5
Systemic treatment	9.0 (7.6–10.5)	31	13	6
RT palliative	4.4 (0.0–13.5)	12.5	12.5	0
**Stage – treated**				
I	62.9 (12.3–113.5)	80	75	54
II	22.4 (7.9–36.9)	64	45.5	27
III	15.1 (8.2–22.1)	59	35.5	25.5
IV	9.0 (7.6–10.4)	31.5	13	5

RT: radiotherapy

In the delayed diagnostic group (defined as > 85 days from symptoms to diagnosis), patients were more frequently diagnosed in incurable stages IIIB–IV as compared to the non-delayed diagnostic group (defined as <85 days from symptoms to diagnosis). The difference was statistically significant (Table 3).

**Table 3:** Early-stage (IA–IIIA) versus late-stage (IIIB–IV) based upon time in days from symptoms to diagnosis, divided by the median of 85 days. More patients were diagnosed in late-stage NSCLC in the diagnostic delayed group. The difference was statistically significant (*p* = 0.04, 2-sided Fishers exact test).

Variables	< 85 days, *n* (%)	> 85 days, *n* (%)	Total, *n* (%)
Stage IA-IIIA	35 (23)	20 (13)	55 (36)
Stage IIIB-IV	45 (29)	54 (35)	99 (64)
Total	80 (52)	74 (48)	154 (100)

### Mycobacterium tuberculosis

A total of 153 patients (94%) were tested for TB by either QFT, sputum analysis or both. QFT testing was done in 120 (74%) of which 32 (27%) were tested positive and/or by sputum analyses in 142 (87%) of which three (2%) were tested positive. All three patients who tested positive by sputum were also tested positive by QFT.

## Discussion

We identified 232 lung cancer patients, encompassing NSCLC, SCLC, and patients without pathological confirmation, corresponding to an annual incidence of 39. For NSCLC only, the incidence was 33 (Supplementary Table 1). In the 2015 study, the incidence was 29 for histologically verified NSCLC and SCLC [14]. In contrast, data from the Association of the Nordic Cancer Registries (2017–2021) shows a slightly higher annual incidence of 51, which is likely due to differences in study periods, diagnostic criteria, or reporting practices [4].

The younger age of median 65 years at diagnosis in Greenland, as compared to 71–72 years in Denmark, may be partly due to smoking patterns [15]. Daily smoking is reported for 13% in Denmark [16] and, unchanged, for 52% in Greenland [17]. Smoking habits may also influence the distribution of histological subtypes, with squamous cell carcinoma remaining the predominant type. Stage at diagnosis remains a significant concern in Greenland. Even though more patients were diagnosed in early-stage NSCLC (IA–IIIA) in 2020 (34%) as compared to 2015 (30%), a substantial proportion of patients (61%) were diagnosed at advanced stages (IIIB–IV), unchanged since 2015 (Supplementary Table 2). However, there has been a decrease in late-stage adenocarcinomas (78%) from 2015 to 2020 (61%). Late-stage diagnosis in Denmark has remained relatively constant at 56 to 58% [15]. The Danish numbers include all lung cancer histologies and are therefore not fully compatible with our selected cohort of NSCLC.

The proportion of patients receiving treatment has remained relatively unchanged since the 2015 study. Over the years, however, there has been a notable increase in treatments with curative intent. Specifically, 18% of patients underwent primary surgery as compared to 12% in 2015. Three patients treated with primary palliative RT had early-stage disease but for different patient-related reasons, curative treatment was not an option. Fifteen per cent did not receive treatment after diagnosis. The majority of these patients had PS 3–4, potentially indicating the impact of late diagnosis and/or comorbidity [18]. Significant comorbidity may have an impact on the fraction of patients receiving oncological treatment; however, we did not assess this retrospectively.

The introduction of ICI in 2023 has expanded treatment options, although their impact on OS is yet to be fully realized. Two patients received ICI before 2023, as the patients were diagnosed and started on treatment when in Denmark. The ICI was discontinued when the patients returned to Greenland. ICI treatment is based on programmed death ligand 1 (PD-L1) status. PDL-1 analysis was not routinely performed in the earlier years; for example, only four patients had PD-L1 analysis done in 2015, increasing to 29 in 2020. Twenty-one patients had PD-L1 >1%, stage IIIB-IV and PS of 0–1, therefore potentially eligible for ICI today. However, comorbidity can further opt out some patients.

Survival rates have shown some improvement. The mOS of 11.2 months for treated patients is comparable to the 2015 data [14]. However, stage-specific survival has improved for stages I, III, and IV. The 1-year survival rate for treated patients was 48%, lower than the 55% observed in Danish patients in 2020 and may partly be explained by the higher fraction of untreated patients in Greenland. But the Greenlandic 2020 1-year survival rate was also lower as compared to the 2015 study, where 58% of treated patients were alive after 1 year. This decrease may be explained by the inclusion of palliative RT in the current study. Patients treated with surgery or long fractionated RT, with or without chemotherapy, had 1-year survival rates of 86 and 77%, respectively. Comparable data from the 2015 study are not available. For Danish patients in 2020, the corresponding 1-year survival rates were 93 and 84%, respectively. While this shows a slight difference in survival rates, it may be influenced by various factors such as healthcare systems or patient demographics. For patients receiving palliative systemic treatment and palliative radiotherapy, the 1-year survival rates were 31 and 13%, respectively. In comparison, Danish patients treated with either palliative chemotherapy or RT had a 1-year survival rate of 43%. The difference could be due to several factors, for example, difference in PS as well as greater opportunities for supportive care within the Danish healthcare system.

Upfront molecular analyses are increasingly performed in the initial diagnostic workflow. Only nine patients in total had molecular analyses performed in 2015 and 2016. The number increased to 26 in 2019 and 2020 with the majority in late stage. The introduction of genomic analyses has improved treatment options for NSCLC patients that harbor specific genomic targetable alterations [19–21]. The frequencies of the targetable *EGFR* mutation and ALK fusion are both comparable with European data. Hence, onco-driven NSCLC in Greenland is molecularly profiled in the same setup as Danish patients.

The median time from symptom onset to diagnosis was 85 days, significantly longer than the 21 days reported in Denmark and confirms one of the main conclusions from the report of Evaluation of the Greenlandic Cancer Strategy 2013 [22]. This delay is influenced by several factors, as discussed below. TB is endemic in Greenland, and TB testing is done in all patients undergoing diagnostic procedures for lung cancer at QIH. It may be difficult to distinguish active or previous TB from new tumors on a chest X-ray, which delay further examination with a CT scan. Furthermore, access to a CT scan is restricted and can only be performed at QIH. As TB testing is done in all patients undergoing diagnostic procedures for NSCLC at QIH, we did not perform statistical analyses for TB testing and diagnostic delay. Scarcity in healthcare personnel in the towns and settlements along the coast also adds time to diagnostics. Previously, the diagnostic bronchoscopy was performed by consulting medical doctors at QIH. Today, local medical doctors have gained expertise to perform the procedure, which is expected to decrease the diagnostic delay.

Overall, diagnostic delay complicates the timely identification and treatment of NSCLC, and the high proportion of late-stage diagnoses and untreated patients continues to negatively impact OS rates in the Greenlandic cohort. To improve NSCLC outcomes in Greenland, several strategies should be implemented. Increasing access to advanced diagnostic tools, such as CT scans and PET scans, and continuous training of local medical staff to perform specialized procedures can reduce diagnostic delays. Developing clear referral pathways and action cards for healthcare personnel in remote areas can ensure prompt diagnostic work-up and reduce delays. Combining TB and lung cancer screening protocols can help differentiate between the two conditions and expedite appropriate diagnostic procedures [23].

## Conclusion

While NSCLC survival in Greenland has improved over the past decade, significant challenges remain. The trend towards diagnosing more stage IA–IIIA patients, and the recent improvements in diagnostic and therapeutic options in Greenland, are expected to translate into a better prognosis in the coming years. Addressing diagnostic delays and enhancing treatment options are crucial steps towards improving outcomes for NSCLC patients in Greenland.

## Supplementary Material

Clinico-pathological characteristics and treatment outcome in non-small cell lung cancer in Greenland 2015–2020 – a comparison with the cohort from 2004 to 2010

## Data Availability

An approval for publication of the database was not included in the project approval from the Greenlandic Ethical Committee nor the hospital management of QIH. Therefore, data from the study are not publicly available, but part of the database can be made available upon request to the corresponding author.
