# Supplementary material for: Clinico-pathological characteristics and treatment outcome in non-small cell lung cancer in Greenland 2015–2020 – a comparison with the cohort from 2004 to 2010
Source: Acta Oncol. 2024 Dec 17;63:41078. doi: 10.2340/1651-226X.2024.41078 (PMC11681137; doi:10.2340/1651-226X.2024.41078)
Supplement: Clinico-pathological characteristics and treatment outcome in non-small cell lung cancer in Greenland 2015–2020 – a comparison with the cohort from 2004 to 2010 [file AO-63-41078-s1.pdf]

Supplementary material has been published as submitted. It has not been copyedited, or typeset by Acta Oncologica

Supplementary table 1. Incidence of newly diagnosed patients with NSCLC, and the number of patients not receiving treatment. There was a slight increase in the incidence in NSCLC and a higher percentage of patients not receiving treatment in the study period.

Year	Newly diagnosed NSCLC	Patients not receiving treatment (% of total diagnosed that year)
2015	20	2 (10.0)
2016	23	5 (21.7)
2017	27	1 (3.7)
2018	35	3 (8.6)
2019	27	6 (22.2)
2020	31	8 (25.8)

Supplementary table 2. Comparative overview in the period of 2015 to 2020 of median overall survival, %-diagnosed stages, and %-histologies in Greenland. The 2015 data are based on the article by Gelvan et al, 2015 (14).

Greenland	2015	2020
Stage, median OS (months)		
- I	56.5	62.9
- II	55.4	22.4
- III	12.1	15.1
- IV	5.8	9.0
Stage, % diagnosed		
- I-IIIA	30%	34%
- IIIB-IV	62%	61%
- NA	8%	5%
Histology, %		
- Adenocarcinoma	35%	36%
- Squamous cell carcinoma	57%	61%
- NA	8%	3%
